# A Missense Mutation in the Vacuolar Protein Sorting 11 (*VPS11*) Gene Is Associated with Neuroaxonal Dystrophy in Rottweiler Dogs

**DOI:** 10.1534/g3.118.200376

**Published:** 2018-06-26

**Authors:** Katherine L. Lucot, Peter J. Dickinson, Carrie J. Finno, Tamer A. Mansour, Anna Letko, Katherine M. Minor, James R. Mickelson, Cord Drögemüller, C. Titus Brown, Danika L. Bannasch

**Affiliations:** * Departments of Population Health and Reproduction, School of Veterinary Medicine, University of California, Davis, Davis, CA 95616; † Surgical and Radiological Sciences, School of Veterinary Medicine, University of California, Davis, Davis, CA 95616; ‡ Department of Clinical Research and Veterinary Public Health, University of Bern, 3001 Bern, Switzerland; § Department of Veterinary and Biomedical Sciences, College of Veterinary Medicine, University of Minnesota, Minneapolis, MN 55455

**Keywords:** autophagy, canine, lysosome, neurodegenerative, inherited, genetic

## Abstract

Canine neuroaxonal dystrophy (NAD) is a recessive, degenerative neurological disease of young adult Rottweiler dogs (*Canis lupus familiaris*) characterized pathologically by axonal spheroids primarily targeting sensory axon terminals. A genome-wide association study of seven Rottweilers affected with NAD and 42 controls revealed a significantly associated region on canine chromosome 5 (CFA 5). Homozygosity within the associated region narrowed the critical interval to a 4.46 Mb haplotype (CFA5:11.28 Mb – 15.75 Mb; CanFam3.1) that associated with the phenotype. Whole-genome sequencing of two histopathologically confirmed canine NAD cases and 98 dogs unaffected with NAD revealed a homozygous missense mutation within the Vacuolar Protein Sorting 11 (*VPS11*) gene (g.14777774T > C; p.H835R) that was associated with the phenotype. These findings present the opportunity for an antemortem test for confirming NAD in Rottweilers where the allele frequency was estimated at 2.3%. *VPS11* mutations have been associated with a degenerative leukoencephalopathy in humans, and *VSP11* should additionally be included as a candidate gene for unexplained cases of human NAD.

Neuroaxonal dystrophy (NAD) is a relatively non-specific histopathological diagnosis for a group of neurodegenerative disorders characterized by dystrophic changes of the neuron followed by development of axonal swellings or spheroids (Revesz *et al.* 2015). First described by Cajal, (Cajal 1928) axonal swellings may occur in the central or peripheral nervous system and the underlying pathogenesis of the variable structured material found in these swellings is often poorly defined. The dystrophic phenotype may vary depending on age of onset, clinical manifestations, and specific disorder.

NAD can be divided into three major etiological groups: *physiological*, *secondary*, and *primary*. *Physiological* NAD can be seen commonly in humans and domesticated species as a component of aging (Suzuki *et al.* 1979; Saito 1980; Summers *et al.* 1995; Borràs *et al.* 1999; Gavier-Widen *et al.* 2001; Revesz *et al.* 2015), and *secondary* NAD may be seen focally, or more widely throughout the nervous system in response to a wide variety of conditions, including trauma, infection, toxin exposure and metabolic disease such as vitamin E deficiency or organophosphate exposure (Yagishita 1978; Summers *et al.* 1995; Revesz *et al.* 2015). Axonal spheroids have also been described in human patients with amyotrophic lateral sclerosis, Alzheimer’s, Parkinson’s disease and hereditary spastic paraparesis. *Primary* NAD is generally associated with a group of genetically heterogeneous, inherited neurodegenerative diseases where the presence of neuroaxonal dystrophy is a major pathological component of the disease. In human patients, neuroaxonal dystrophic pathology has been associated to varying degrees with several genetically defined disease syndromes, most prominently in Infantile Neuroaxonal Dystrophy (INAD) and Pantothanate Kinase-associated Neurodegeneration associated with alterations in the *PLA2G6*, and *PANK2* genes respectively. Many of the human NAD syndromes are also associated with brain iron accumulation, including alterations in the *PLA2G6*, *PANK2*, *FTL*, *C19orf12*, *FA2H*, and *WDR45* genes (Revesz *et al.* 2015; Arber *et al.* 2016). Neuroaxonal dystrophy in humans without iron accumulation is seen in Wilson’s disease and Nasu-Hakola disease involving the *ATP7B* and *DAP12/TREM2* genes respectively, and in “neuroaxonal leukoencephalopathy with axonal spheroids” which has no defined genetic cause to date (Revesz *et al.* 2015). *Primary* NAD has been reported in most domesticated species including dogs (Clark *et al.* 1982; Chrisman *et al.* 1984; Blakemore and Palmer 1985; Evans *et al.* 1988; Sacre *et al.* 1993; Franklin *et al.* 1995; Bennett and Clarke 1997; Siso *et al.* 2001; Nibe *et al.* 2007; Fyfe *et al.* 2010; Fyfe *et al.* 2011; Hahn *et al.* 2015; Pintus *et al.* 2016; Degl’Innocenti *et al.* 2017; Tsuboi *et al.* 2017), cats (Woodard *et al.* 1974; Carmichael *et al.* 1993; Rodriguez *et al.* 1996; Résibois and Poncelet 2004), cattle (Hanshaw *et al.* 2015), sheep (Cordy *et al.* 1967; Nuttall 1988; Harper and Morton 1991; Kessell *et al.* 2012; Finnie *et al.* 2014), pigeons (Barrows *et al.* 2017), mice (Bouley *et al.* 2006), and horses (Beech 1984; Blythe *et al.* 1991; Aleman *et al.* 2011; Finno *et al.* 2013; Finno *et al.* 2016), where an association with vitamin E deficiency, along with a genetic susceptibility, has been reported.

In dogs, breed related NAD has been reported as fetal onset in Giant Schnauzer-Beagle mix breed dogs (Fyfe *et al.* 2010; Fyfe *et al.* 2011), juvenile onset in Dachshund mix breed dogs (Pintus *et al.* 2016), Border collies (Clark *et al.* 1982), Chihuahuas (Blakemore and Palmer 1985; Degl’Innocenti *et al.* 2017), Jack Russell Terriers (Sacre *et al.* 1993), Papillons (Franklin *et al.* 1995; Nibe *et al.* 2007; Tsuboi *et al.* 2017), Spanish Water Dogs (Hahn *et al.* 2015), and young adult or adult onset in Rottweilers (Cork *et al.* 1983; Chrisman *et al.* 1984; Evans *et al.* 1988; Bennett and Clarke 1997; Siso *et al.* 2001) and English Cocker Spaniels (McLellan *et al.* 2003). NAD in Cocker Spaniels is accompanied by retinal degeneration and is associated with vitamin E deficiency (McLellan *et al.* 2003). Specific genetic mutations associated with the *PLA2G6*, *TECPR2* and *MFN2* genes have been identified in the Papillon, Spanish Water Dog and Schnauzer-Beagle cross dogs respectively (Fyfe *et al.* 2011; Hahn *et al.* 2015; Tsuboi *et al.* 2017).

Rottweiler NAD was first reported in the early 1980s and is characterized by a young adult age of onset with mild progression of clinical signs, typically including postural deficits, ataxia, hypermetria, intention tremor and nystagmus (Cork *et al.* 1983; Chrisman *et al.* 1984). Clinical signs reflect the predominantly sensory topographical distribution of pathology within the central nervous system (CNS) consisting of mild cerebellar atrophy, large numbers of axonal spheroids, and demyelination of axons in the vestibular nucleus, lateral and medial geniculate nuclei, sensory nucleus of the trigeminal nerve, gracilis and cuneate nuclei, and in the spinal cord dorsal horn (Cork *et al.* 1983; Chrisman *et al.* 1984). Based on a small pedigree, it was hypothesized to be an autosomal recessive disorder (Cork *et al.* 1983).

Defining underlying genetic mechanisms for breed related neuroaxonal dystrophies in dogs has the potential to provide biological insight and potential translational models for the heterogeneous disease phenotypes seen in human patients (Shearin and Ostrander 2010; Hytönen and Lohi 2016). A genome-wide association was therefore performed with samples from the original four reported Rottweiler cases (Cork *et al.* 1983; Chrisman *et al.* 1984) and three additional cases, together with whole-genome sequencing of selected cases to identify candidate genes for NAD in Rottweilers.

## Materials and Methods

### Canine Samples

Buccal swabs or blood samples were collected from privately owned dogs through the William R. Pritchard Veterinary Medical Teaching Hospital at UC Davis. Collection of canine blood samples was approved by the University of California, Davis Animal Care and Use Committee (protocol #16892). Additional Rottweiler DNA samples were provided by the University of Minnesota and the University of Bern, Switzerland. These studies were approved according to the national guidelines for animal welfare by the Institutional Animal Care and Use Committees (IACUC) of the University of Minnesota, and by the Cantonal Committee for Animal Experiments (Canton of Bern; permits 23/10, 48/13 and 75/16) for the University of Bern. Owners specified the breed of each dog. Genomic DNA was extracted using the Qiagen kit (QIAGEN, Valencia, CA). Neurological phenotypes were determined by a veterinarian and confirmed postmortem via necropsy when available.

### Genome-wide SNP Genotyping

Genome-wide SNP genotyping was performed on seven cases and 42 controls, using the Illumina CanineHD 220k BeadChip (Illumina, San Diego, CA, USA). All samples had a genotyping rate of ≥ 90%. 62,193 SNPs were excluded due to a minor allele frequency ≤ 5% and 7,421 SNPs were excluded due to a high genotype failure rate (≥ 10%), leaving 151,799 SNPs after quality control. A Chi-square analysis and a genomic inflation factor (λ) was calculated with PLINK (Purcell *et al.* 2007). Homozygosity throughout the associated interval was analyzed by visual inspection assisted by color-coding homozygous genotypes in Excel. Homozygosity in the affected dogs, that passed the Bonferroni threshold (*P* ≤ 3.29×10^−7^), was used to narrow down the regions of association and was visualized using Haploview (Barrett *et al.* 2005; Barrett 2009; Clarke *et al.* 2011). Figures were made in R using the ggplot2 package (Wickham 2009).

### Whole-Genome Sequencing

Whole-genome sequencing was performed as described on 100 canine genomes, (Brown *et al.* 2017) with two histopathologically confirmed Rottweiler cases and 98 dogs unaffected with NAD, across 25 different breeds, including two Rottweilers. Sequencing was performed on the Illumina HiSeq 2000 using 100bp paired-end reads with approximately 8.7x coverage per sample. The reads were aligned to the canine reference genome (CanFam3.1) (Lindblad-Toh *et al.* 2005). Local realignment and variant calls were performed using the Genome Analysis Tool Kit (GATK version 2.5-2gf57256b) pipeline (McKenna *et al.* 2010). Biological consequences of variants were predicted using Ensembl’s Variant Effect Predictor (VEP), PolyPhen-2 (v2.2.2r398), and SIFT (Adzhubei *et al.* 2010; Sievers *et al.* 2011; Sim *et al.* 2012; Adzhubei *et al.* 2013; McLaren *et al.* 2016;).

### Genotyping by Sanger Sequencing

Primers were designed using Primer3 (Rozen and Skaletsky 2000) to validate the putative functional mutation uncovered in *VPS11* (F: CTGCAGGTCCCTGTCCTAAG; R: TGTACCTGGCTCTTGGCTCT). PCR products were sequenced using the Big Dye termination kit on an ABI 3100 Genetic Analyzer (Applied Biosystems, Foster City, CA). Sequences were evaluated using Chromas (Technelysium, South Brisbane, QLD, Australia). Sequences were aligned to CanFam3.1 using BLAT (UCSC Genome Browser). Allele frequency was calculated excluding the seven affected cases.

### RNA Extraction and cDNA Sequencing

RNA was isolated from liver using Qiagen QIAamp Blood Mini Kit tissue protocols (QIAGEN, Valencia, CA). RNA was reverse transcribed into cDNA using Qiagen QuantiTect Reverse Transcription Kit. Ubiquitously expressed *VPS11* (F: TGGTCCAAAAACTGCAGAAA; R: CTCAAAGCAGTGTTGGTGGA) and the housekeeping gene *RPS5* (Brinkhof *et al.* 2006) cDNA were PCR amplified from liver tissue from two affected Rottweilers, one Gordon Setter, and one mixed breed dog. *RPS5* was amplified in liver to ensure equivalent amounts of cDNA were produced. The PCR products were sequenced on an ABI 3500 Genetic Analyzer and analyzed using Chromas (Technelysium, South Brisbane, QLD, Australia). The sequences were aligned to Can Fam3.1 using BLAT (UCSC Genome Browser) to confirm the missense mutation in the cDNA of *VPS11*.

### Data Availability

The SNP genotyping data can be found in files Supplemental 1 (File S1), and Supplemental 2 (File S2). Whole-genome sequencing files reported in this paper can be found in the NCBI Sequence Read Archive (SRA Bioproject no. PRJNA377155). Sequences from four Pugs were made available in 2012 by TGEN (https://www.tgen.org/patients/canine/). Supplemental material available at Figshare: https://doi.org/10.25387/g3.6214010.

## Results

### Case Definition

DNA samples were available from the four original NAD affected Rottweilers from Cork *et al.* (Cork *et al.* 1983) and three additional cases. One case was evaluated at the Veterinary Medical Teaching Hospital VMTH (University of California, Davis) with neurologic deficits and histopathological findings at necropsy consistent with previously reported cases. Blood samples from two additional dogs were submitted by their respective owners. Both dogs were presented to veterinarians with a history and clinical signs consistent with NAD and were noted to be “clumsy” as puppies. One dog was presented at approximately 1 year of age with generalized ataxia and hypermetria and absent menace responses. The second dog was presented for progressive ataxia and hypermetria that had been present for several years. Both dogs were alive and ambulatory at 2 and 5 years of age respectively. The second dog had been tested previously for mutations associated with two other neurodegenerative diseases reported in Rottweilers (leukoencephalomyelopathy (Minor *et al.* 2018), laryngeal paralysis-polyneuropathy (Mhlanga-Mutangadura *et al.* 2016)) and was negative for both mutations.

### Genome-Wide Association Study

**Figure 1:**
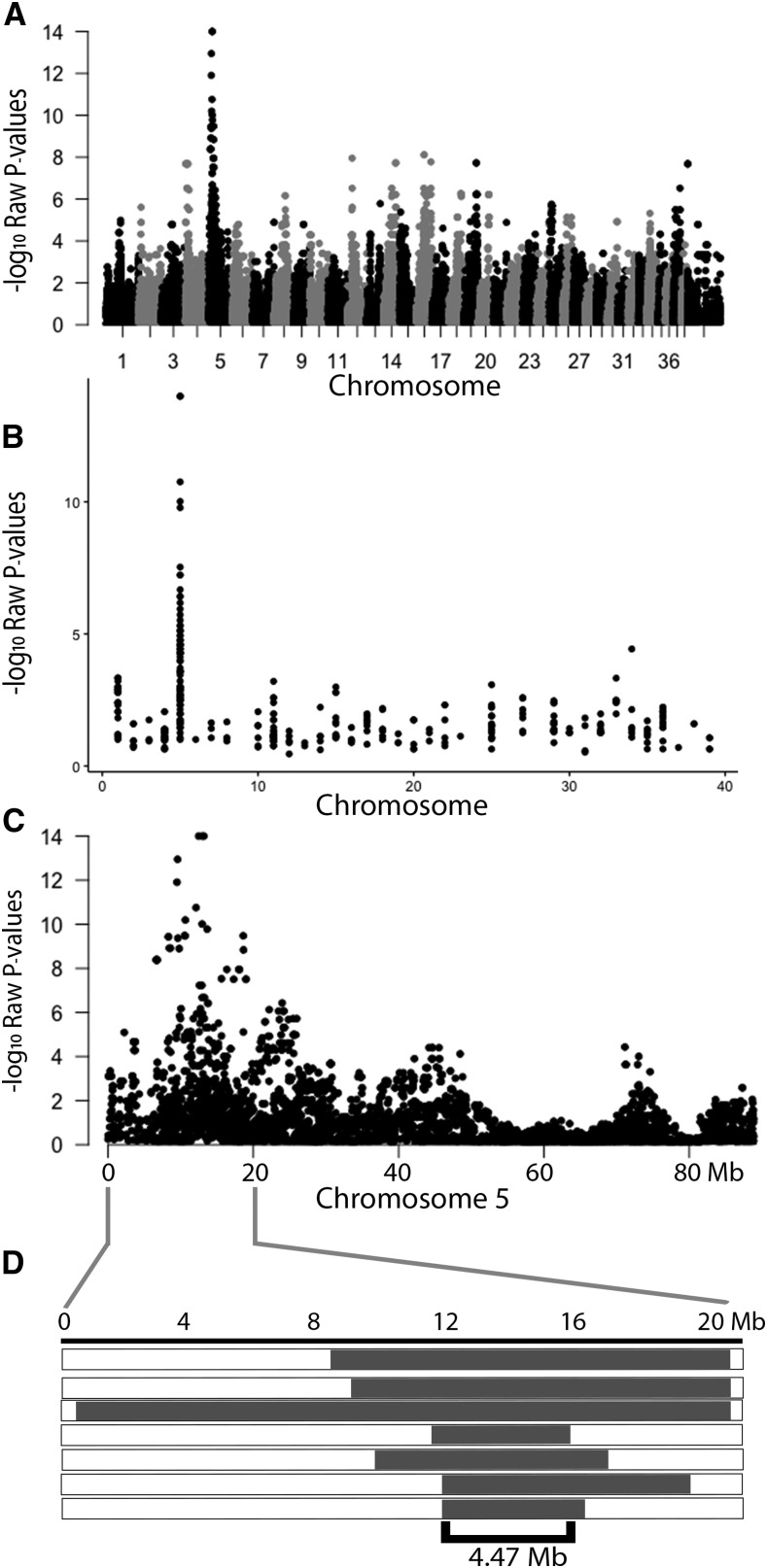
GWAS for Rottweiler NAD. A) Manhattan plot for the NAD GWAS showing the –log10 of the raw P values (Y axis) for each genotyped SNP by chromosome (X axis). Genomic inflation (λ) was 1.52. B) SNPs with an allele frequency of 100% in cases were plotted; with the –log10 of the raw P values (Y axis) for each SNP by chromosome (X axis). C) Plot of the –log10 of the raw P values (Y axis) for each SNP on canine chromosome 5 (CFA 5). D) Haplotypes observed in the seven cases, showing homozygosity throughout the associated region. Runs of homozygosity are marked by the gray horizontal bars. The critical interval is marked by the shared homozygous haplotype in between the black bracket (CFA5: 11.29 Mb – 15.75 Mb).

To identify loci associated with the NAD phenotype in the Rottweiler dog, a genome-wide association study was performed, followed by homozygosity analysis using seven cases affected with NAD and 42 healthy controls. Four of the seven cases were directly related resulting in a genomic inflation (λ) value of 1.52. A chi-square analysis of the 151,799 SNPs, identified preliminary associations on canine chromosomes (CFA) 4, 5, 12, 14, 16, 19, and 37 (Figure 1A). The lowest *P* value was on CFA 5 (*P* = 1×10^−14^) and 26 SNPs in this region were more associated than on the next highest chromosomal location. Since this disease is uncommon and pedigree analysis was consistent with a recessive mode of inheritance, a homozygosity analysis was performed in the cases. To identify regions of homozygosity in the cases, *P* values from markers with an allele frequency of 1 in the cases were plotted. There were 45 markers that met the Bonferroni correction (*P* ≤ 1×10^−4^), and all but one (CFA 34; *P* = 3.71×10^−5^) were on CFA 5, making it the only statistically significant region of association that met the allele frequency criteria (Figure 1A-C). Homozygosity throughout the associated interval was used to narrow down the region of interest to 4.46 Mb (Chr5:11,282,754-15,754,443; CanFam3.1).

### Whole-Genome Sequencing

Variants within the critical interval, identified in the GWAS, were analyzed for association using 98 dogs unaffected with NAD and two Rottweilers histopathologically confirmed to have NAD. Within the critical interval, there were 73 genes, and 31,749 SNPs and 17,421 indels were identified. 164 SNP variants and 15 indels segregated with the phenotype were within the region identified on CFA 5. Only a single SNP was found to be protein coding (CFA 5:14,777,774) among the segregating variants. The remaining variants were 3′ UTR (n = 4), downstream (n = 23), intergenic (n = 74), intronic (n = 56), a non-coding transcript (n = 5), or upstream variants (n = 16).

### VPS11 Non-Synonymous Variant

**Figure 2:**
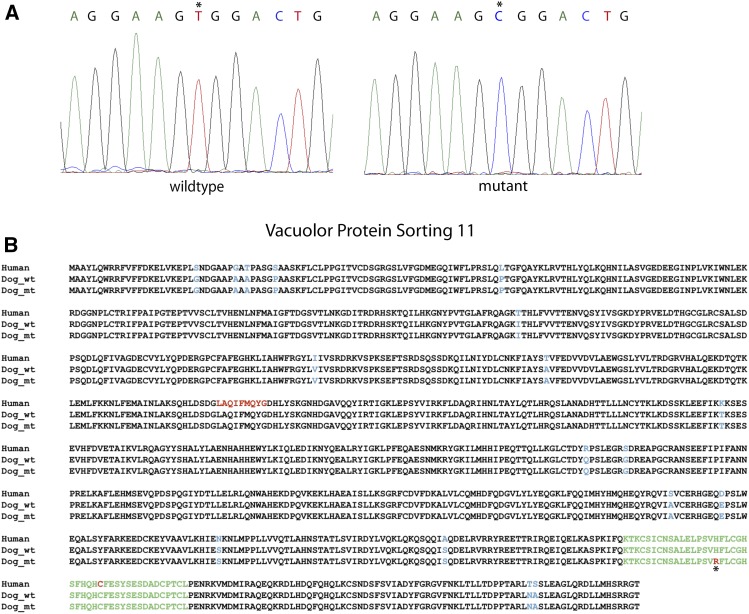
*VPS11* sequence electropherograms and amino acid alignment with human VPS11. A) Electropherogram of the missense mutation (*) (g.14777774T > C) within the cDNA of *VPS11*. B) Amino acid sequence alignment of human and dog (wild type and mutant) Vacuolar Protein Sorting 11 showing 98.2% amino acid conservation across species. The Zinc RING finger domain is in green with the location of the missense variant denoted by an asterisk below the aligned sequence (specific amino acid is highlighted in red). Non-conserved amino acids are in blue, and known disease causing mutations in human patients are in red.

A non-synonymous variant was identified on CFA 5 (g.14777774T > C; CanFam3.1) in the Vacuolar Protein Sorting 11 (*VPS11*) gene. The variant leads to an amino acid change (p.H835R) in the Zinc RING finger domain of the protein (Figure 2B), which is ultimately predicted to be deleterious (VEP: moderate; PolyPhen-2: 0.999; SIFT: 0). The cDNA of *VPS11* was sequenced from liver from two NAD affected Rottweilers, one Gordon Setter, and one mixed breed dog, (Figure 2.A) to confirm the presence of the mutation in the mRNA (*VPS11*c.2504A > G). *VPS11* is highly conserved across species (Table S1), with humans and dogs having 98.2% conservation at the amino acid level (Figure 2.B).

### VPS11 Variant Genotyping

440 dogs, consisting of 288 Rottweilers, and 152 dogs from 19 other breeds, were genotyped for the *VPS11* mutation (Table 1). Of the 288 Rottweilers, 13 were identified as heterozygous for the mutation, seven were homozygous for the mutation (cases as described above), and the remaining 268 Rottweilers, along with the 152 other dogs, were all homozygous for the reference allele. Of the Rottweilers genotyped, 211 were from the United States of America (204 wild type, three heterozygous, and seven homozygous mutants); 75 were from Europe (65 wild type and 10 heterozygous). The frequency of the mutant allele in this population is estimated to be 2.3%.

**Table 1:** *VPS11* (g.14777774T > C) MUTATION GENOTYPING RESULTS

BREED	TOTAL	*VPS11/VPS11*	*VPS11/vps11*	*vps11/vps11*
Boston Terrier	**2**	2	0	0
Boxer	**33**	33	0	0
Brittany	**3**	3	0	0
Bulldog	**5**	5	0	0
Dachshund	**1**	1	0	0
French Bulldog	**3**	3	0	0
German Shorthaired Pointer	**4**	4	0	0
Golden Retriever	**39**	39	0	0
Great Dane	**2**	2	0	0
Irish Setter	**1**	1	0	0
Labrador Retriever	**17**	17	0	0
Mixed Breeds	**6**	6	0	0
Newfoundland	**4**	4	0	0
Nova Scotia Duck Tolling Retriever	**13**	13	0	0
Pug	**5**	5	0	0
Rottweiler	**288**	268	13	7
Saluki	**2**	2	0	0
Weimaraner	**5**	5	0	0
West Highland White Terrier	**2**	2	0	0
Whippet	**5**	5	0	0

## Discussion

Seven Rottweilers that presented with clinical signs consistent with the NAD phenotype were homozygous for a non-synonymous mutation within the RING-finger domain of the *Vacuolar Protein Sorting 11 (VPS11)* gene (Figure 2). In order to overcome significant population stratification based on relatedness of the affected cases, a genome-wide association followed by homozygosity mapping was used. This type of approach has been used successfully in the past to identify breed specific Mendelian recessive diseases in dogs (Drögemüller *et al.* 2009; Kropatsch *et al.* 2010; Forman *et al.* 2016).

**Figure 3:**
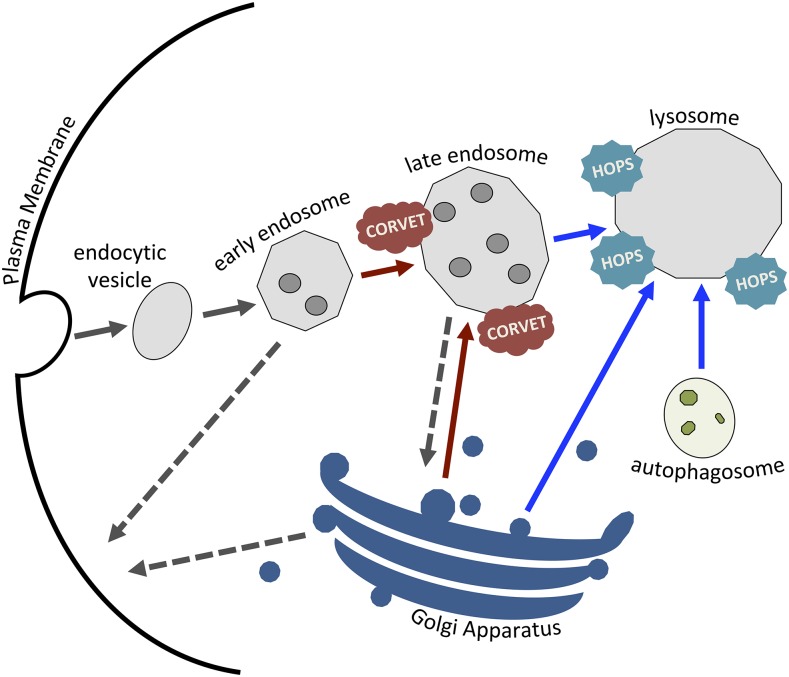
Schematic representation of the endosome-autophagosome-lysosome pathway. VPS11 is a key constituent of the VPS class C complexes CORVET (red) and HOPS (blue). Disruption of the CORVET/HOPS tethering complexes, and subsequently the membrane fusion processes required for appropriate trafficking, would be consistent with both the lysosomal storage and NAD phenotypes seen in human and dog disease, secondary to accumulation of membrane and cytosolic constituents. Blue arrows represent fusion events mediated by HOPS, red arrows represent fusion events mediated by CORVET, dashed gray arrows represent pathways of exocytosis and plain gray lines represent pathways of endocytosis.

Sorting and degradation of internalized cell surface proteins and lipids in eukaryotic cells is controlled through the “endocytic network” (Balderhaar and Ungermann 2013; Spang 2016), such that surface proteins may progress through early and late endosomes before they are degraded in lysosomes, or may be sorted and recycled. Disposal and recycling of cytoplasmic components is similarly achieved through the autophagosome-lysosome pathway during autophagy (Levine and Klionsky 2004; Nixon 2013) (Figure 3).

Two VPS class C complexes, CORVET and HOPS, each composed of multiple different VPS proteins are essential for control of the membrane fusion machinery and trafficking of material through these endosome-lysosome organelles. CORVET and HOPS act as tethers, in coordination with other key proteins such as RAB5 and RAB7, and bring appropriately targeted vesicles into close proximity with the target membrane (Richardson *et al.* 2004; Balderhaar and Ungermann 2013; Perini *et al.* 2014; van der Kant *et al.* 2015; Spang 2016). Both CORVET (class C core vacuole/endosome tethering complex) and HOPS (homotypic fusion and protein transport) contain a four-subunit core consisting of VPS11 (PEP5), VPS16, VPS18 (PEP3), and VPS33, which are conserved across yeast, insects, plants, and mammals (Nickerson *et al.* 2009). VPS11 has been shown to have a key role in determining selective binding to either early or late endosomes, and as an integrator of the complex assembly (Plemel *et al.* 2011; van der Kant *et al.* 2015). The RING domain of VPS11 that harbors the non-synonymous mutation in Rottweiler NAD has been shown to be important specifically in fusion at the vacuole (lysosome) in yeast (Plemel *et al.* 2011).

Mutation of the *VPS11* gene in humans is associated with an infantile onset neurological syndrome characterized by hypomyelination and variable neurological deficits including motor and cognitive impairment, dystonia, ataxia, visual deficits, and seizures (Figure 2.B) (Edvardson *et al.* 2015; Hörtnagel *et al.* 2016; Zhang *et al.* 2016). Histopathological characterization has not been done. However, the syndrome is classified as a leukoencephalopathy based on MRI (magnetic resonance imaging) findings, and skin and bone marrow biopsies were suggestive of a lysosomal storage type disease (Hörtnagel *et al.* 2016). Consistent with the known function of VSP11 (Plemel *et al.* 2011), *in vitro* studies of the mutant human protein resulted in disruption of late endosome/vacuole fusion and the autophagic pathway (Edvardson *et al.* 2015; Zhang *et al.* 2016). Although the Rottweiler *VPS11* mutation is in a similar location to one of the documented human mutations within the VPS11 RING finger domain (Figure 2.B) (Edvardson *et al.* 2015; Zhang *et al.* 2016), the clinical phenotypes appear to have distinct differences, most notably the apparent white matter *vs.* gray matter distribution of lesions in humans *vs.* dogs. The human *VPS11* syndrome also appears to be characterized by lysosomal accumulations compared to axonal spheroids (Hörtnagel *et al.* 2016), although detailed histopathological characterization of human CNS lesions is not available. However, this spectrum of intracellular accumulations of varying types is within the rational consequences of disruption of the endosome-autophagosome-lysosome system predicted following VPS11 (CORVET/HOPS) dysfunction. Additionally, species and site specific differences in pathological phenotype for mutations within the same gene are well documented across a broad range of genetic diseases. For example, a variety of clinical and pathological phenotypes have been reported for alterations within the same genes that cause some of the neuroaxonal dystrophy syndromes in humans (Revesz *et al.* 2015; Arber *et al.* 2016); *PLA2G6* gene mutations can give a spectrum of disease phenotypes as well as classical INAD including dystonia-parkinsonism syndromes and spastic paraplegia (Gregory *et al.* 2008; Ozes *et al.* 2017). Similarly, alterations in the *MFN2* gene give rise to fetal onset NAD in dogs (Fyfe *et al.* 2010; Fyfe *et al.* 2011); however, human alterations result in peripheral nervous system syndromes (Charcot-Marie-Tooth disease type 2A2 and hereditary motor and sensory neuropathy type 6A (Del Bo *et al.* 2008)), while mouse knockouts and transgenic overexpression and cattle with altered *MFN2* have CNS and PNS neurodegeneration but do not have NAD (Drögemüller *et al.* 2011). Protein site-specific effects, species differences in pathological responses (such as lack of iron accumulation in canine NAD), and differences in gain or loss of function mutations may all contribute to the phenotypic heterogeneity.

Despite this heterogeneity within the NAD disease phenotype, common pathological pathways are implicated in many NAD syndromes, including the primary and secondary diseases. Vitamin E deficiency has been associated to varying degrees with axonal dystrophy in both experimental and clinical settings, in several species including dogs, rodents, horses and primates (Nelson *et al.* 1981; Pillai *et al.* 1994; McLellan *et al.* 2003; Finno *et al.* 2013; Finno *et al.* 2016). The importance of the complex relationship between pathways controlling reactive oxygen species (ROS) and autophagy has been well documented (Underwood *et al.* 2010; Fang *et al.* 2017), and the autophagy pathway is particularly important in the context of the highly metabolic neuron (Nixon 2013). Previously defined genes associated with NAD in dogs (*PLA2G6*, *TECPR2* and *MFN2*) as well as many human NAD related genes have been proposed as potential modulators of the autophagy pathway (Fyfe *et al.* 2011; Hahn *et al.* 2015; Meyer *et al.* 2015; Arber *et al.* 2016; Tsuboi *et al.* 2017), and the currently described *VPS11* gene alteration in Rottweiler NAD would be predicted to affect the autophagic, as well as other lysosomal pathways. High conservation of *VPS11* between species, the essential role *VPS11* plays in the endosomal-autophagy-lysosomal pathways, and the impact of mutations in *VPS11* leading to neurodegenerative diseases, provides strong support for the missense mutation identified in Rottweiler NAD to be causative for the disease and a potential candidate for unexplained forms of human NAD. Detailed biological analysis of the Rottweiler *VPS11* specific mutation will be needed to fully understand the apparent species/mutation differences in disease expression and its potential value as a translational model.
